# Case of Mr. William Windsor, of Bettesfield, in the Parish of Harweir, and County of Flint, a Farmer's Son, Ten Years of Age

**Published:** 1801-03

**Authors:** Thos. Jones

**Affiliations:** No. 51, Strand


### *To the Editors of the Medical and Phyfical Journal.*

Gentlemen,

I Received the following cafe from my friend Mr. Humphrey, of Shrewfbury; if inferted in the Medical Journal, it will afford much fatisfaction to Your moft humble fervant,

*No.* 51, *Strand, Dec.* 17, 1800. THOS. JONES.

---

### *Cafe of Mr. William Windfor, of Bettesfield, in the parifh of Harweir, and county of Flint, a farmer's son, ten years of age.*

About four years ago the cornea of the left eye appeared flat, and of a green colour, with the entire lofs of fight; but continued free from pain until laft Chriftmas, when the colour became black, and the eye preferved its former fhape, but became very painful. About Whitfuntide there appeared a fmall opening in the cornea, through which was difcharged a tea fpoonful of bloody matter; and in the courfe of a fortnight after, a fungus began to appear through the aperture, which grew very flowly for fome time, being kept down by the eye-lids; in the beginning of Auguft it had grown beyond the eye-lids, and was increafing very faft; at the latter end of which month he came to Shrewfbury, where I firft faw him with my partner Mr. Sandford, and we agreed to remove it; but were obliged to poftpone the operation until Sept. 19, at which time it was increafed to double the fize it was when I firft faw him, and began to be floughy in the middle, attended with bleeding every time it was dreffed. From the increafed fize of it he was in conftant pain except when lying upon his back, as it covered the whole of the cheek. I paffed the knife between the nofe and the tumor, as it extended too far over the cheek to allow me to get at the root without wounding the eye-lids on the other fide; the hæmorrhage from it was not fo great as we expected, and was ftopped by preffure alone. He became eafy in a fhort time after the operation, and has continued fo fince, without any unpleafant fymptom. He was always greatly difpofed to drowfinefs, and has a large head. Upon examining the tumor, the excrefcence appeared to grow from the retina, which was become a pulpy mafs, filling the whole cavity of the eye. The tumor weighed ℥vfs. and was fo reflected over the eye as to prefs upon the orbit, and completely envelope the eye, which was forced out of its focket, and the mufcles put upon the ftretch fo much as to increafe increase their thickness, although their action continued. The optic nerve (which was divided) was perfectly sound, the tunica albuginea preserved its healthy state, although the cornea was diseased to its edge. The part was entirely healed by the 11th of October, when he left Shrewsbury perfectly well, and has continued so ever since.

I regret not having procured a drawing of it, and I never saw any case similar, except in Heister's Surgery; and I had no idea of publishing it for some time after the operation.

